# The research on enhancing the strength and durability of pipeline connection ports using arc welding additive manufacturing

**DOI:** 10.1371/journal.pone.0324598

**Published:** 2025-06-04

**Authors:** Xiaoben Chen, Bo Zhang, Fanglin Shen, Chunxiao Jiao, Jingshu Chang, Chuan Feng, Wei Li

**Affiliations:** 1 Construction Project Management Branch, National Petroleum and Natural Gas Pipeline Network Group Co., Ltd., Langfang, China; 2 School of Mechanical Engineering, University of Science and Technology Beijing, Beijing, China; Manipal Academy of Higher Education, INDIA

## Abstract

In long-distance pipelines, the connection between pipelines is often the weakest link. During the manufacturing process of pipelines, roundness errors or defects are inevitable, and the greater the difference between the maximum and minimum diameters, the greater the impact on the strength of pipeline connections. At present, the main method to improve the pipe end is casting upsetting. However, there are currently no reports on using additive manufacturing technology to optimize pipeline ellipticity and improve pipe end strength and stiffness. This article solves the problem of weak weld strength at the connection end of oil pipelines and proposes for the first time the method of using arc additive manufacturing technology to thicken and reinforce the pipe end. By analyzing the ellipticity of the pipe end, arc based additive manufacturing technology is used to thicken the pipe end, thereby improving the strength and stiffness of the pipeline connection weld. This paper establishes a mathematical model for optimizing the design of pipeline connection ends, with remanufacturing thickness, length, and pipe end radius as design variables and pipe end strength and stiffness as constraints, and obtains the optimal parameter combination. The optimization results show that under typical working conditions, the optimized pipe end strength is significantly improved, the strain is significantly reduced, and the pipeline can withstand higher pressure, thereby improving the overall reliability and safety of the pipeline.

## 1. Introduction

Long-distance pipelines are essential for transporting resources such as oil and natural gas. With the continuous expansion of pipeline networks, the connection points, particularly welded joints, have become one of the weakest links in pipeline systems [1,2]. The quality and reliability of pipeline connections directly impact the pipeline’s overall safety and economic performance. This is particularly true when there are common manufacturing errors, such as roundness deviations and diameter inconsistencies. These geometric imperfections often lead to stress concentration at the connection points, affecting the pipeline’s strength and durability [3,4]. Traditional methods for strengthening pipeline connections typically involve welding or machining to thicken the pipe ends, thereby improving the strength and stiffness of the connection points [5,6]. However, these methods often suffer from drawbacks such as complex processes, high costs, and difficulties in controlling geometric shapes. This is particularly problematic when small shape defects at the connection points lead to stress concentration, potentially causing localized damage or failure [7]. To enhance the performance of pipeline connections and reduce stress concentration issues, additive manufacturing (AM) technology, especially Arc Welding Additive Manufacturing (AWAM), has emerged as a promising solution in recent years [8,9]. AWAM technology uses an electric arc to melt metal and build up the target structure layer by layer, offering advantages such as high efficiency, flexibility, and lower costs. AWAM not only effectively repairs and enhances the mechanical properties of metal components but also allows for precise control over the geometry of the repaired areas [10,11]. Recently, AWAM has made significant advancements in metal component repair and pipeline reinforcement [12,13]. For example, recent studies have shown that AWAM can precisely optimize the shape at pipeline connection points, reducing stress concentration and improving the overall strength and durability of the pipeline [14,15]. Although the application of AWAM technology in pipeline repair has gained widespread attention, research on optimizing the geometry of pipeline connection ends and enhancing their strength and stiffness through the rational design of additive manufacturing parameters remains limited [16,17]. Systematic studies on the optimization design of pipe end geometries, such as pipe end ellipticity, thickening thickness, length, and corner shapes, have yet to form a complete theoretical framework and optimization method [18]. Many emerging research findings have applied AWAM technology in pipeline connection points. For instance, Wang et al. [19]. Explored the impact of deviations in outer diameter and wall thickness on pipelines Hou et al. [20] analyzed the influence of geometric discontinuities in pipelines on the mechanical properties of joints Qin et al. [21] studied the influence of geometric defects on gas pipelines. Chen et al. [22] studied the repair effect of AWAM technology on wear-resistant pipelines, emphasizing the critical role of geometric shape optimization in reducing stress concentration. Li et al. [23] analyzed the potential of AWAM technology to repair complex pipeline structures, suggesting that it can effectively enhance the strength and durability of pipelines. Recent studies have highlighted the potential of additive manufacturing to enhance material performance in various sectors. Jatti et al. [24] explored Laser Powder Bed Fusion to optimize the mechanical properties of superalloys, demonstrating the advantages of AM in improving material strength. Similarly, Nandy et al. [25] developed a cost-effective, open-source micro-tensile tester using 3D printing, which offers precise material testing capabilities. Zhou et al. [26] conducted numerical simulations to study the application of AWAM at pipeline connection points, showing that precise geometric shape control can significantly improve the strength of the pipeline. Although these studies have proposed various AWAM optimization methods to some extent, most of them focus on parameter adjustments during the repair process, while overlooking the comprehensive impact of pipeline connection geometry (such as ellipticity, thickness distribution, and corner shapes) on pipeline performance. Additionally, Zhang et al. [27] proposed a pipeline shape optimization method based on AWAM technology, improving the mechanical performance of pipeline connection points and reducing welding defects. Wu et al. [28] explored the advantages of combining AWAM technology with traditional repair methods, suggesting that this combination can effectively enhance the overall quality of pipeline repairs. Yang et al. [29] introduced an AWAM process optimization method incorporating machine learning for pipeline repair design. Yang’s results show that his method significantly improves the reliability of repaired pipelines. Chen et al. [30] studied the impact of AWAM process parameters on pipeline weld joints, indicating that optimized process parameters can improve the welding quality at pipeline connection points. However, these studies mostly focus on applying different repair methods or parameter combinations, lacking a systematic analysis and investigation into the optimization of pipe end geometry. In particular, the consideration of design variables such as pipe end ellipticity, thickening thickness, length, and corner shapes has not been adequately explored in existing research.

This study proposes a novel optimization approach for pipe end remanufacturing based on AWAM technology, aiming to address the gap in the existing literature. Specifically, it introduces a refined geometric design and optimization strategy that targets common stress concentration issues, thereby significantly enhancing the overall mechanical performance of pipeline connection points. Furthermore, the study integrates an analysis of pipe end ellipticity with the AWAM process, which allows for the precise thickening and reinforcement of pipe joints, ultimately improving their strength and stiffness. A mathematical model is developed to optimize the pipe joint design, incorporating strength and stiffness as the key constraint conditions. This model takes important design parameters, such as reinforcement thickness, length, and the shape and size of the fillets, into account. After applying this model, the optimization method improves mechanical performance substantially, compared to traditional thickening methods. Ultimately, by focusing on both the optimization of additive manufacturing parameters and the geometry of the pipe ends, this study provides valuable insights and lays a solid theoretical foundation for the future application of AWAM technology in pipeline repair and design.

## 2. The effect of pipe roundness on welding

### 2.1. Analysis of the influence of roundness on welding and related standards

The ellipticity of steel pipes is a critical factor affecting their visual quality and functional performance. It directly influences the weld seam’s geometry, dimensions, and stress distribution. Poor roundness can result in localized stress concentration, increasing the risk of crack formation during the welding. This issue is especially critical in high-pressure transmission pipelines, where excessive ellipticity can weaken the pipes’ strength and reduce their durability. Under external loads and pressure, excessive ellipticity can lead to stress concentration and crack propagation.

Excessive ellipticity can lead to uneven stress distribution in pipes, potentially causing localized stress concentration within the pipeline. Non-compliance with standard ellipticity specifications in steel pipes during the welding can lead to defects such as misalignment, cracking, and residual stress formation. These defects significantly affect the quality of the weld seam and the service life of the pipeline. Under external loads and pressure, the strength and durability of steel pipes may be severely compromised [31,32].

According to Section 4.2 of the *Structural Steel Pipe Manufacturing Technical Specifications* (4th Edition, 1990), the allowable roundness error for steel pipes is explicitly defined as follows:

(1) For steel pipes with a wall thickness of 50.8 mm (2 inches) or less, the maximum roundness error must not exceed 1% of the nominal diameter or one-quarter of the maximum outer diameter.
(2) For steel pipes with a wall thickness greater than 50.8 mm, the ratio of roundness error to wall thickness must not exceed 1:8.

These standards provide essential technical requirements for steel pipes, ensuring stability in performance during subsequent processing and use. Strict roundness control is necessary to improve production quality and is also a critical guarantee that steel pipes meet design requirements of structural safety, pressure resistance, and durability.

### 2.2. Definition and impact of ellipticity in the study

In this study, the ellipticity of the steel pipe is defined as the difference between the maximum diameter ($d_{\max}$) and the minimum diameter ($d_{\min}$) of the pipe cross-section, as shown in the following formula:


$$\Delta =d_{\max}-d_{\min}$$ (1)

The impact of ellipticity on welding is primarily reflected in the following aspects:

(1) Welding quality. The ellipticity of steel pipes and variations in diameter are critical and directly affect the quality of on-site welding. Steel pipes with lower ellipticity ensure a more uniform stress distribution under high-pressure service conditions, thereby enhancing the strength of the weld joints and maintaining the overall integrity of the pipeline [33].
(2) Pipe end misalignment. Ellipticity directly influences the misalignment at the pipe ends during alignment. Ellipticity exceeding the standard can lead to welding defects, such as cracks and residual stresses, which negatively affect the welding quality [34].
(3) The impact of sizing machine on ellipticity correction. After forming, the pipe blank’s ellipticity significantly impacts the steel pipe’s final ellipticity after correction by the sizing machine. This, in turn, greatly influences geometric errors during the welding process [35].

### 2.3. Mechanical performance analysis and simulation study

**Fig 1:**
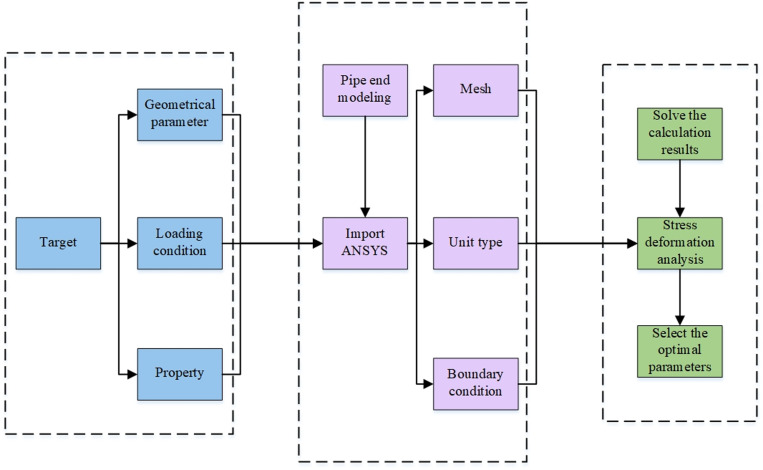
Flow chart of pipe end simulation analysis.

To further analyze the impact of ellipticity on the performance of steel pipes and provide a foundation for subsequent remanufacturing, this study used Ansys Workbench software to analyze the mechanical performance and simulate the deformation of pipes with varying ovalities. The ANSYS software version used in this simulation is 2022R1. For the material selection of the pipeline, ordinary alloy steel with a yield strength of 345Mpa and a tensile strength of 550Mpa was chosen, with an elastic modulus of 206Gpa, Poisson’s ratio of 0.3, and a yield ratio of 0.7. The simulation results, which considered stress distribution and deformation under different ellipticity conditions, revealed that minor changes in ellipticity result in relatively small variations in stress and deformation. However, when ellipticity differences are significant (e.g., an ellipticity of 10), the variations in stress and deformation become substantial, the simulation flowchart for overall analysis is shown in Fig 1.

Specific analysis results are as follows:

(1) Ellipticity is 0 (Standard Circular Pipe)

**Fig 2:**
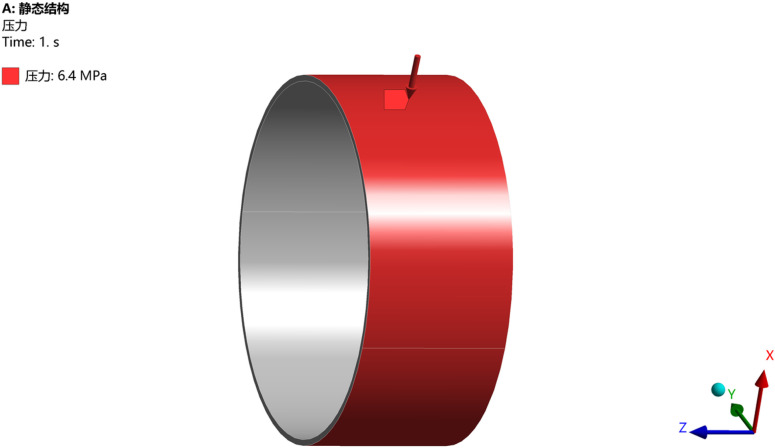
Load application diagram for pipeline with ellipticity of 0.

**Fig 3:**
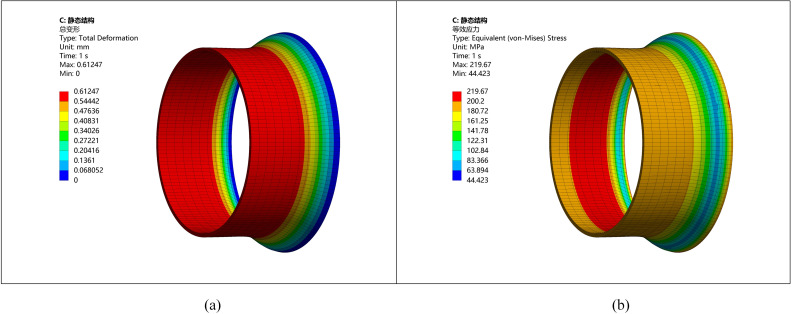
Stress and strain distribution diagram of pipeline with ellipticity of 0. (a) Stress diagram of pipeline (b) Strain diagram of pipeline.

When the ellipticity is 0, meaning the pipe is a standard circular shape, the selected diameter is 1219 mm with a wall thickness of 20 mm. The load is applied on the outer wall of the pipe, and the constraint surface is set at the tail end of the pipe. The load application diagram is shown in Fig 2, and the stress distribution and deformation diagram obtained from simulation analysis are shown in Fig 3:

In the stress distribution diagram, the stress is 6.4 MPa, and Fig 3a illustrates the uniform distribution of stress on the outer wall of the pipe. In Fig 3b, the deformation is minimal, and the pipe maintains a stable shape.

(2) Ellipticity is 3

**Fig 4:**
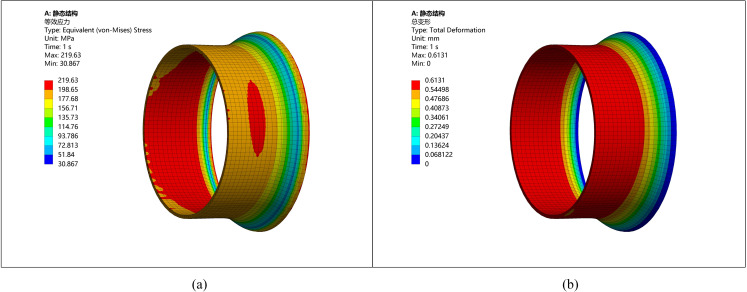
Stress strain diagram of pipeline with ellipticity 3. (a) Stress diagram of ellipticity 3 (b) Deformation diagram of ellipticity 3.

When the ellipticity is 3 mm, other parameters remain unchanged. When the ellipticity changes too little, it will not have a significant impact on the overall stress-strain of the pipeline. And the difference from unbiased pipelines is very small. The stress and deformation diagrams with an ellipticity of 3 mm are shown in Fig 4:

(3) Ellipticity is 5

**Fig 5:**
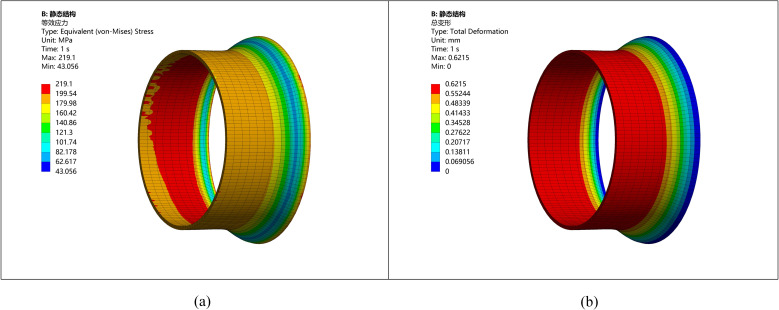
Stress strain diagram of pipeline with ellipticity 5. (a) Stress diagram of ellipticity 5 (b) Deformation diagram of ellipticity 5.

When the ellipticity is 5 mm, the difference between the maximum and minimum diameters is 5 mm. All other parameters remain the same as in the model with an ellipticity of 0, with only the ellipticity being altered. Simulation analysis shows that with an ellipticity of 5, the changes in stress distribution and deformation become more noticeable. The specific stress distribution and deformation diagrams are shown in Fig 5.

(4) Ellipticity of 10

**Fig 6:**
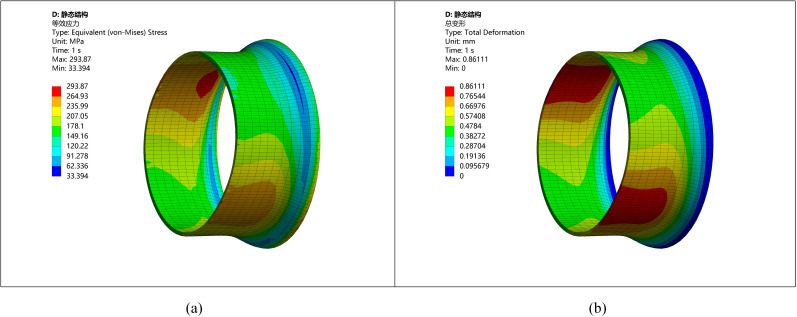
Stress strain diagram of pipeline with ellipticity 10. (a) Stress diagram of ellipticity 10 (b) Deformation diagram of ellipticity 10.

When the ellipticity is 10 mm, the steel pipe exhibits considerable deviations, which significantly impact welding quality. The simulation results demonstrate that pipes with greater ellipticity display uneven stress distribution, which may lead to severe local stress concentrations and deformation. The stress distribution and deformation diagrams are presented in Fig 6.

Based on the above analysis and simulation results, ellipticity significantly impacts the stress distribution and deformation of the pipeline. Therefore, controlling ellipticity is essential to ensure welding quality and the operational performance of the pipe. However, within a small range of ellipticity variation (e.g., when the ellipticity difference is less than 3 mm) the differences in stress and deformation are negligible. Only when the ellipticity difference ranges from 3 to 5 mm the changes become more noticeable.

## 3. Pipe end remanufacturing process optimization model

### 3.1. Analysis of pipeline interface improvement using arc welding additive manufacturing technology

In previous engineering projects, most of the efforts to increase the thickness and strength of pipeline ports were made through the use of pipe head upsetting technology. However, this requires high thermal deformation ability of materials, and the processing cost of some materials that are not easily deformed will increase. Using arc additive manufacturing technology to improve pipe segment ports will be more flexible and targeted. AWAM achieves precise thickening of complex geometric shapes and high material utilization by layer stacking of metal materials through electric arcs. In addition, AWAM can improve the microstructure and enhance the mechanical properties of pipe ends, such as strength and toughness, by optimizing process parameters. In contrast, the traditional upsetting method achieves thickening of the pipe end through plastic deformation, resulting in a dense structure and higher strength. However, the upsetting method requires high plasticity of the material, making it difficult to achieve thickening of complex shapes, and may result in performance differences due to uneven deformation. Therefore, AWAM has more advantages in complex shapes and high-performance customization requirements, and can purposefully reinforce weak areas, saving material costs.

### 3.2. Design variables and constraints

In the pipe end thickening design, taking a pipeline with a diameter of 1219 mm as an example, the optimization focuses on design variables such as the thickening length, thickening height, and the fillet radius of the transition area. The thickening height, length, and transition fillet radius are key design parameters. Analytical optimization is conducted on these parameters to improve the repair quality and pipeline performance.

During the optimization design process, the following constraints are set:

(1) The thickening height at the pipe end must not exceed the pipeline wall thickness.
(2) Considerations for ellipticity correction, machining allowance compensation (usually 2–3 mm), additive manufacturing errors, and stress improvement.
(3) The remanufacturing length is generally not to exceed 1000 mm, as exceeding this length reduces the effectiveness of stress improvement and significantly increases costs.

By optimizing these constraints, the stress and deformation of the remanufactured pipeline are minimized, ensuring its performance and long-term stability.

### 3.3. Optimization Objective Function and Evaluation Formula

An objective function is established to ensure that the stress and deformation of the remanufactured pipeline are minimized. Based on linear elastic fracture mechanics theory, the following evaluation formula (2) is used to estimate the performance of the pipeline:


$$S_{r}^{\prime }=\frac{\left(SF_{m}\right)\sigma_{m}}{\sigma_{y}-\frac{-\pi \sigma_{b}}{8\sigma_{m}}{\left[({\frac{\pi \sigma_{b}}{8\sigma_{m}})}^{2}+1\right]}^{\frac{1}{2}}\Gamma_{m}}$$ (2)

In the formula, $SF_{m}$ is the safety factor, $\sigma_{m}$ is the membrane stress, $\sigma_{y}$is the material yield strength, $\sigma_{b}$ is the bending stress, $R_{0}$ and $R_{i}$ are the outer and inner diameters of the pipeline, respectively, and $\Gamma_{m}$ is the stress distribution function,$R_{c}$ is the distance between the crack tip and the center of the pipeline, with the calculation formula as follows [36]:


$$\Gamma_{m}=\frac{R_{0}^{2}-R_{c}^{2}+\left(1-\frac{\theta }{\pi }\mathrm{\backslash rightleft}(R_{c}^{2}-R_{i}^{2}\right)}{R_{0}^{2}-R_{i}^{2}}$$ (3)

Based on formula (2), the strength and performance of the pipeline under different repair conditions can be effectively evaluated. Formula (4) evaluates the safety of the X80 steel base material and weld, according to the SY/T6477 standard.

For the X80 base material, the calculation is performed using formula (4):


$$K_{r}=\frac{1.75}{1+\exp\left(\frac{L_{r}-1.32}{0.19}\right)}-0.77,L_{r}\leq L_{r}^{\max}$$ (4)

For the X80 weld, calculation is performed using formula (5):


$$K_{r}=\frac{206.8}{1+\exp\left(\frac{L_{r}-2.24}{0.19}\right)}-205.82,L_{r}\leq L_{r}^{\max}$$ (5)

These evaluation formulas work efficiently for the safety assessment of welds and pipeline base materials in real-world engineering applications.

### 3.4. Membrane stress analysis

In the pipeline stress analysis, membrane stress is the primary type of stress. Membrane stress can be divided into two components: One is the membrane stress caused by the internal pressure of the pipeline (which is decomposed into axial stress and hoop stress), and the other is the axial membrane stress caused by temperature differences during the transportation process. The axial membrane stress includes the stress $\sigma_{H}$ caused by internal pressure changes and the stress $\sigma_{T}$ caused by temperature differences. The calculation formulas are as follows:


$$\sigma_{H}=\frac{PD}{2t}$$ (6)

$$\sigma_{T}=E\alpha \left(t_{1}-t_{2}\right)$$ (7)

Where P is the internal pressure of the pipeline, D is the outer diameter of the pipeline, t is the wall thickness of the pipeline, α is the material’s coefficient of linear thermal expansion, EE is the material’s elastic modulus, and $t_{1}$ and $t_{2}$ are the temperatures at the two ends of the pipeline. For X80 steel pipelines, take 2.03 × 105 MPa as the elastic modulus EE and 1.2 × 10 − 5 mm/°C as the material’s linear expansion coefficient.

After remanufacturing and repair of the pipeline, assuming no misalignment defects during the welding process, it is only affected by membrane stress. At this point, the membrane stress consists of the membrane stress $\sigma_{m1}$ caused by internal pressure and membrane stress $\sigma_{m2}$ caused by other loads, which are calculated as follows:


$$\sigma_{m1}=\frac{PR_{0}^{2}}{R_{0}^{2}-R_{i}^{2}}$$ (8)

$$\sigma_{m2}=\frac{F}{\pi \left(R_{0}^{2}-R_{i}^{2}\right)}$$ (9)

Where F is the axial force at the center of the pipeline cross-section, P is the internal pressure of the pipeline, and $R_{0}$ and $R_{i}$ are the outer and inner radii of the pipeline, respectively. Finally, the following formula (10) is used to verify whether the pipeline meets the stress requirements:


$$\sqrt{{\sigma_{m1}}^{2}+{\sigma_{m2}}^{2}}\leq [\sigma ]$$ (10)

In this study, the X80 steel pipeline has a wall thickness of 20 mm, a diameter of 1219 mm, and an internal pressure of 6.4 MPa. By replacing the known data, the pipeline stress can be ensured to be less than its allowable number.

## 4. Pipe end remanufacturing process optimization method

### 4.1. Introduction to the optimization of pipe end remanufacturing processes

Pipe end remanufacturing utilizes additive manufacturing technology to repair and rectify the connection between the pipe and flange, focusing on correcting ellipticity and improving mechanical performance. This process includes thickening the pipe ends to ensure a more uniform geometry and performing surface treatments to guarantee high surface quality. The thickening approach varies depending on the connection method of the steel pipes. The methods can be classified based on changes to the internal and external diameters of the pipe, as described below:

(1) Internal Thickening, this method involves reducing the internal diameter of the pipe to increase the wall thickness.
(2) External Thickening, this approach expands the external diameter of the pipe to achieve thickening.
(3) Internal-External Thickening, this method reduces the internal diameter and increases the external diameter, modifying the pipe thickness in both directions.

The comparison of these thickening methods is summarized in Table 1.

**Table 1:** The comparison of these thickening methods.

Factors	Internal Thickening	External Thickening	Internal-External Thickening
**Outer Diameter**	Unchanged	Increased	Increased
**Inner Diameter**	Decreased	Unchanged	Decreased
**Ease of Forming**	Relatively Easy	Easy	Difficult
**Stability of Thickening**	Good	Fair	Poor

### 4.2. Calculation of length shrinkage and deformation resistance during thickening process

The length shrinkage of the steel pipe during the thickening process is a crucial parameter in the remanufacturing process. Once the dimensions of the inner and outer molds are established, the external diameter, internal diameter, and transition zone dimensions of the thickened components become fixed. The length of the thickened section, however, must be designed according to the standards and user requirements. Therefore, the process parameters should be determined based on the principle of volume conservation.

The volume of the shrinkage during the thickening process is given by:


$$\Delta v_{1}=\pi \frac{\left(D^{2}-d^{2}\right)}{4}\cdot \Delta L=\pi (d+\Delta t)\cdot \Delta t\cdot \Delta L$$ (11)

Here D and d represent the outer and inner diameters, and Δt is the thickening height.

After thickening, the increase in volume at the thickened pipe end relative to a flat-ended pipe has two parts in formula (12) and formula (13).

Volume Increase due to the Transition Zone:


$$\Delta v_{g}=\frac{\pi }{3}\left(({\frac{D}{2})}^{2}+\frac{Dd}{4}+{\left(\frac{d}{2}\right)}^{2}\right)L_{g}-\pi {\left(\frac{d}{2}\right)}^{2}L_{g}=\frac{\pi }{3}\Delta t^{2}\cdot L_{g}+\frac{\pi }{2}d\cdot \Delta t\cdot L_{g}$$ (12)

Volume Increase due to the Thickened Pipe Section:


$$\Delta v_{j}=\frac{\pi }{4}D^{2}\cdot L_{j}-\frac{\pi }{4}d^{2}\cdot L_{j}=\pi (d+\Delta t)\cdot \Delta t\cdot L_{j}$$ (13)

The heating length is primarily determined by the length of the deformed section and the transition layer region. The heating temperature varies with the type of steel, typically ranging between 700°C and 1250°C.

Using an approximation of stress-strain behavior during the thickening process, the forging force (FF) can be derived as follows:

The unit forging force P is proportional to the friction coefficient μ and the material’s high-temperature deformation resistance τ:


$$P=K\cdot \mu \cdot \tau_{T}$$ (14)

For actual thickening, assuming the friction coefficient remains constant, the forging force F is expressed as:


$$F=K\cdot \tau_{T}\cdot S$$ (15)

K is a coefficient that depends on the hydraulic forging cylinder area and punch area, and the value of K ranges from 1.1 to 2.0, determined empirically.

High-temperature deformation resistance of metals is a fundamental concept in metal hot working. This resistance describes the ability of the metal to resist plastic deformation when subjected to external forces. It directly affects various aspects of metal processing, including force calculations, energy consumption, process parameter settings, and designing pressure equipment. The high-temperature deformation resistance τ of steel can be modeled by the Arrhenius equation:


$$\tau_{T}=\tau_{0}\exp\left(\frac{A}{T_{K}}\right)$$ (16)

Here A is a constant dependent on the steel composition, and T_k_ is the deformation temperature.

## 5. Influence of process parameters

The thickening height is corrected according to the pipe’s ellipticity. For different ellipticity values, the required thickening height varies. Factors such as flange dimensions, ellipticity correction, machining allowance, and additive manufacturing errors must be considered when selecting a thickening method (external thickening, internal-external thickening, etc.).

### 5.1. Ellipticity correction for a pipe with 5 mm ellipticity

For the simulation analysis of pipeline end correction, it is necessary to divide the appropriate grid, select the appropriate internal pressure, and confirm the fixed support. In this simulation analysis, the grid partitioning method selected multiple regions with a size of 15 mm. The selection of pipeline internal pressure refers to the ASME B31.8 pipeline standard. In actual simulation, 1.6 times the maximum design pressure of the gas distribution pipeline is selected for analysis, which is 6.4Mpa. The fixed support is selected at the bottom of the pipeline.

**Fig 7:**
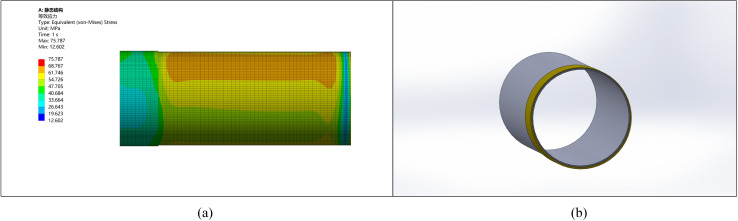
Stress comparison between the thickened area and other parts. (a) Pipeline stress diagram (b) Pipeline model diagram.

A pipe with a diameter of 1219 mm and an ellipticity of 5 mm has a minimum diameter of 1214 mm. Considering ellipticity correction, machining allowance, manufacturing errors, and the improvement of pipe end stress, an initial thickening height of 10 mm and external thickening are chosen, as shown in Fig 7.

### 5.2. Ellipticity correction for a pipe with 10 mm ellipticity

**Fig 8:**
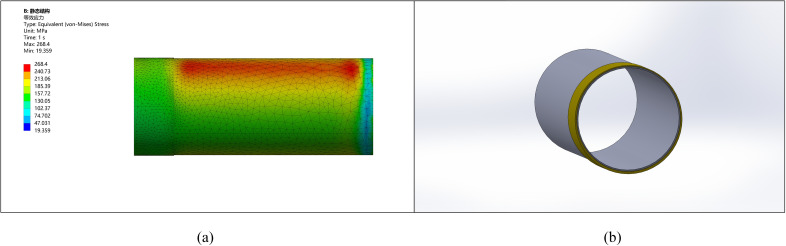
Comparison between the corrected pipe end with an ellipticity of 10 and other parts. (a) Pipeline stress diagram (b) Pipeline model diagram.

Similarly, for a pipe with an ellipticity of 10 mm, after considering correction, machining allowance, and stress improvement, the thickening height is selected as 15 mm, and external thickening is applied, as shown in Fig 8.

The comparison of stress with deformation shows that the stress and deformation changes are more pronounced with higher ellipticity. The stress at the pipe end becomes more uniform after the correction, leading to reduced stress concentration and deformation. Without remanufacturing, ellipticity may result in welding defects such as cracking.

### 5.3. Simulation analysis of thickening length

**Fig 9:**
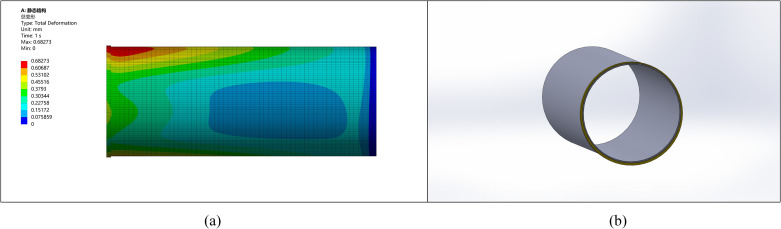
Stress-strain diagram for a pipe with a thickened end length of 50 mm. (a) Pipeline stress diagram (b) Pipeline model diagram.

**Fig 10:**
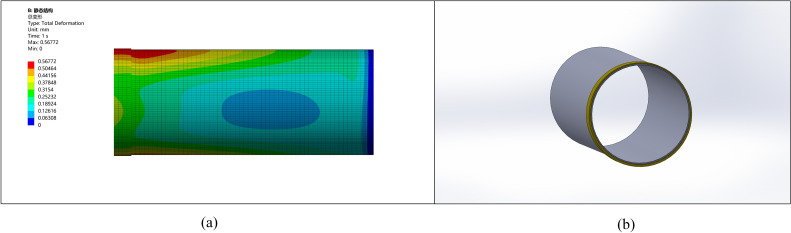
Stress-strain diagram for a pipe with a thickened end length of 200 mm. (a) Pipeline stress diagram (b) Pipeline model diagram.

**Fig 11:**
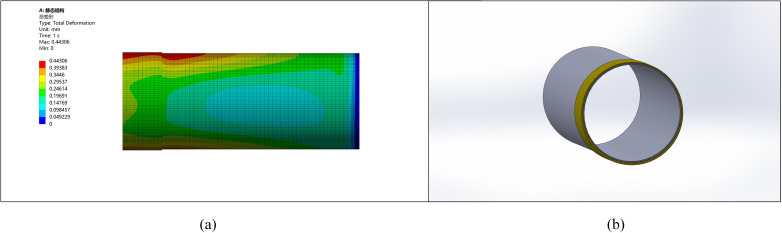
Stress-strain diagram for a pipe with a thickened end length of 500 mm. (a) Pipeline stress diagram (b) Pipeline model diagram.

**Fig 12:**
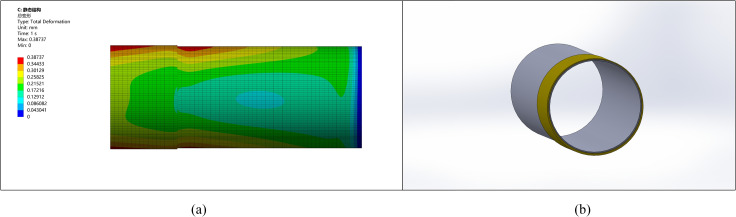
Stress-strain diagram for a pipe with a thickened end length of 800 mm. (a) Pipeline stress diagram (b) Pipeline model diagram.

**Fig 13:**
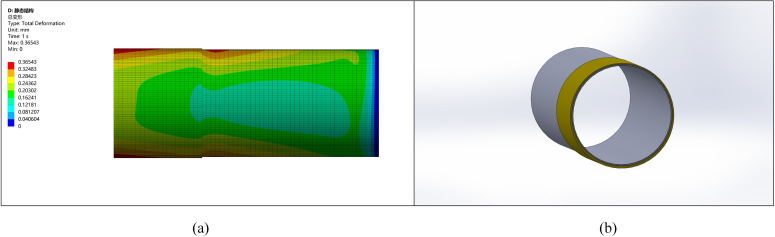
Stress-strain diagram for a pipe with a thickened end length of 1000 mm. (a) Pipeline stress diagram (b) Pipeline model diagram.

**Fig 14:**
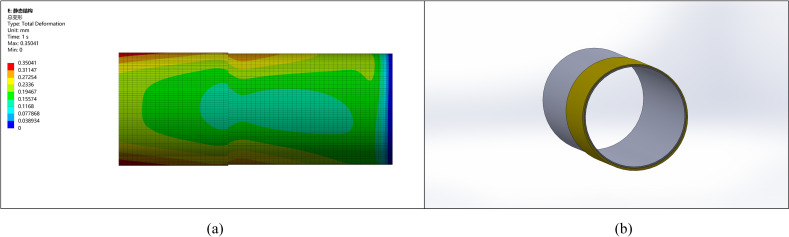
Stress-strain diagram for a pipe with a thickened end length of 1200 mm. (a) Pipeline stress diagram (b) Pipeline model diagram.

The effect of thickening length on pipe end stress and deformation was simulated with a fixed ellipticity of 5 mm and thickening height of 10 mm. Compare several thickening lengths of 50 mm, 200 mm, 500 mm, 800 mm, 1000 mm, and 1200 mm to identify the optimal thickening length data. The stress and deformation graphs for various lengths are shown in Fig 9–14.

It is observed that as the length of thickening increases, stress and deformation decrease, stabilizing at around 1000 mm. This suggests the deformation reaches stability after a thickening length of approximately 1000 mm.

### 5.4. Simulation analysis of transition zone

**Fig 15:**
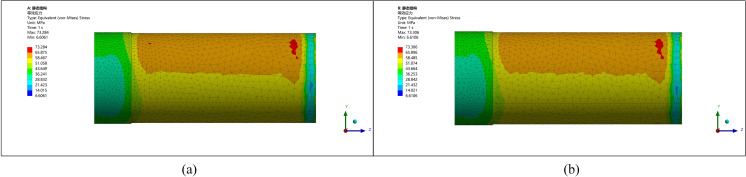
Stress-strain diagram for a radius of 5 mm and 10 mm. (a) Rounding radius 5 mm (b) Rounding radius 10 mm.

**Fig 16:**
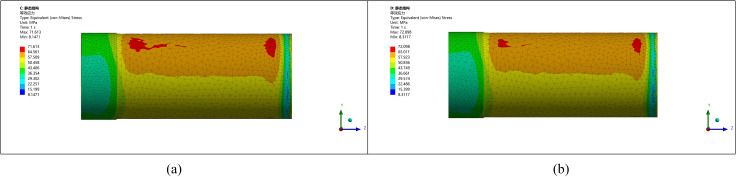
Stress-strain diagram for a radius of 15 mm and 20 mm. (a) Rounding radius 15 mm (b) Rounding radius 20 mm.

Two types of transitions—right-angle and radius—are analyzed to ensure practical and effective implementation. Simulations are conducted for different corner radii (5 mm, 10 mm, 15 mm, 20 mm) at a standard pipe model. The results (Figs 15,16) demonstrate that radius transitions significantly reduce stress concentrations compared to right-angle transitions.

**Fig 17:**
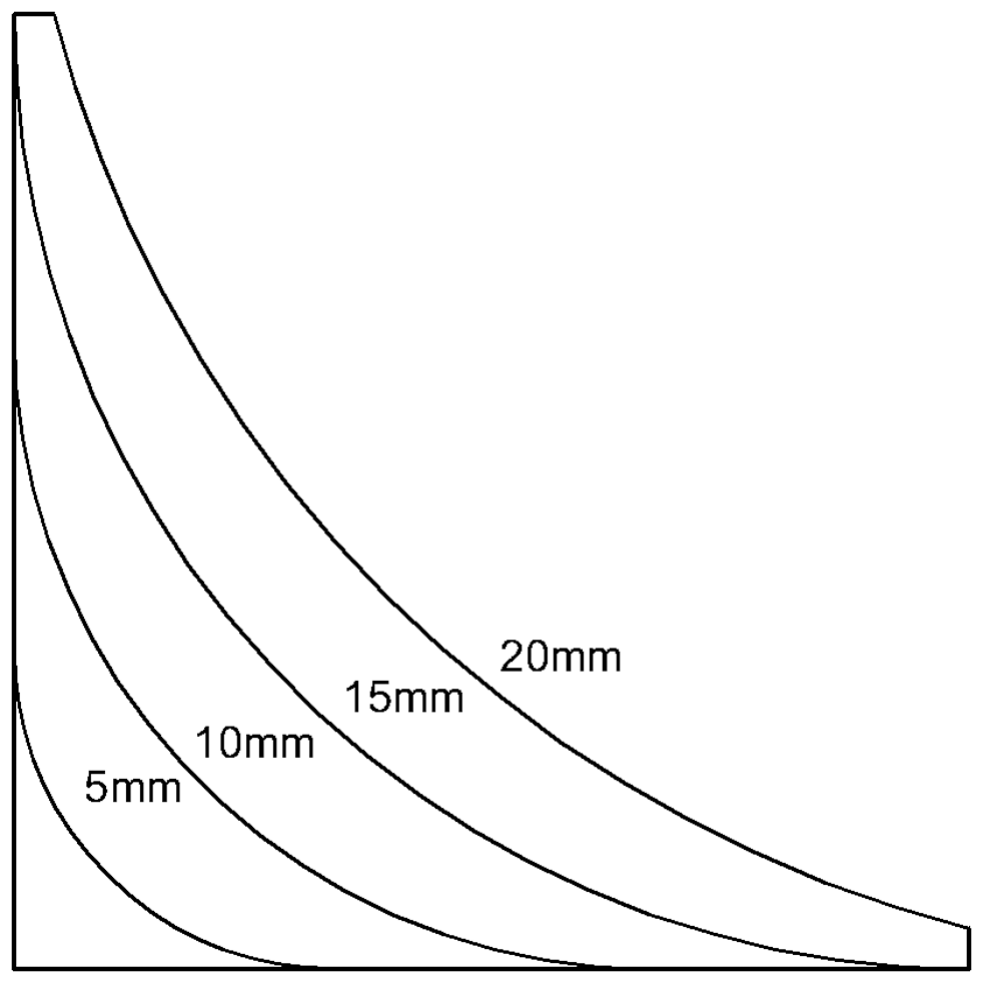
Schematic of the transition layer in pipeline remanufacturing.

Increasing the corner radius further beyond 15 mm leads to a reduction in maximum stress, but beyond 20 mm, stress concentrations increase again due to poor integration of the thickened zone with the original pipe, as shown in Fig 17.

Because the transition layer’s right-angle side is 15 mm, when the right-angle length increases to 15 mm, it no longer effectively satisfies the connection between the remanufactured section and the original pipeline. This leads to an increase in the length of the remanufactured section. Therefore, the right-angle transition should not exceed 10 mm.

**Fig 18:**
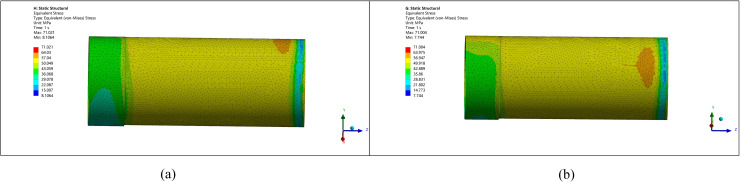
Stress-strain diagrams for right angles of 5 mm and 10 mm. (a) Stress diagrams for right angles of 5 mm (b) Stress diagrams for right angles of 10 mm.

As shown in Fig 18, the right-angle transition experiences greater stress and more significant deformation. Consequently, the probability of failure is higher. From a welding quality perspective, using a radius transition in the transition layer is safer and more reasonable.

**Fig 19:**
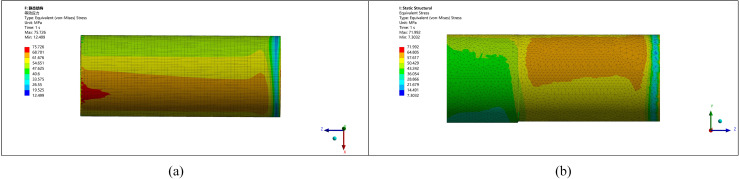
Comparison of pipe ends before and after optimization. (a) Before optimization (b) After optimization.

To integrate the remanufactured length, height, and radius, the optimized simulation analysis is compared with the pre-optimization simulation analysis, as shown in Fig 19 below:

Based on finite element simulations and practical analysis, the optimized remanufacturing parameters are as follows: a thickening height of 10 mm, considering machining allowance, ellipticity correction, and additive manufacturing error; a thickening length of approximately 800 mm where stress changes stabilize; and a radius transition with a corner radius of 15 mm, which offers the best improvement in stress distribution and deformation reduction. This optimized approach significantly enhances welding quality and ensures the structural integrity of the remanufactured pipe end.

### 5.5. Experimental verification

**Fig 20:**
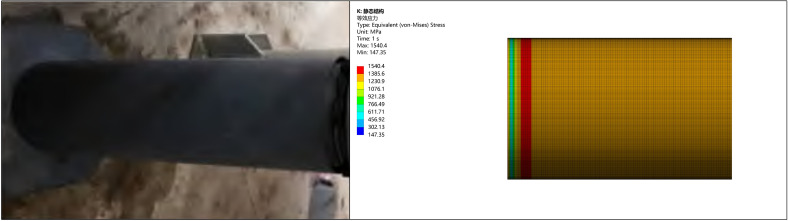
Pipeline test and pipeline simulation diagram.

In order to verify the rationality of the end reinforcement and thickening of additive manufacturing technology, the simulation analysis results were compared and verified with the corresponding pipeline internal pressure experimental test results [37]. In the pressure experiment inside the pipeline, the wall thicknesses were taken as 2.5 mm, 2.7 mm, and 3.0 mm, respectively Conduct ultimate pressure test. The pipeline material is selected as X120 pipeline steel, with a density of ρ = 7850 kg/m3, an elastic modulus of E = 210GPa, a Poisson’s ratio of 0.3, a yield strength of 996MPa, and a tensile strength of 1115MPa. The physical and simulation analysis models of the experimental pipeline are shown in Fig 20.

**Fig 21:**
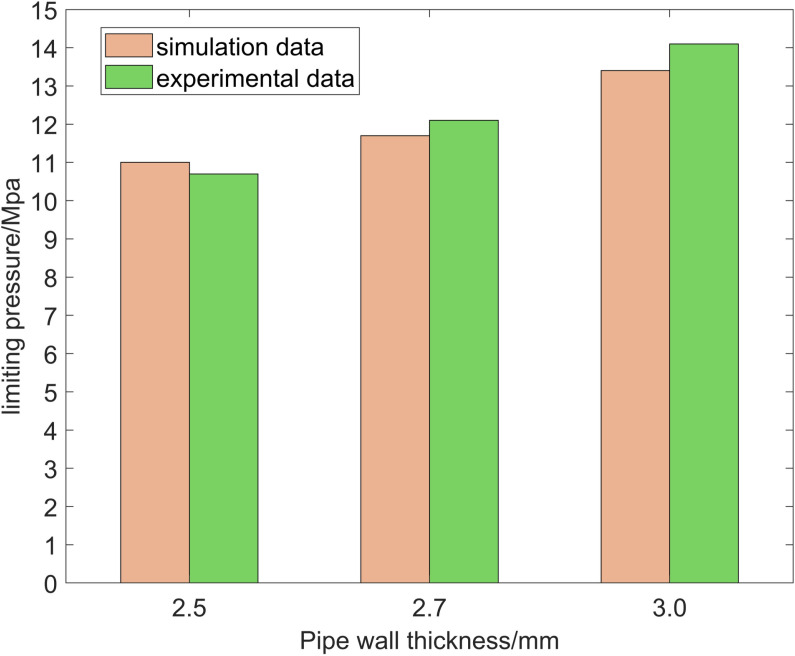
Comparison of simulation and experimental results.

Three real physical prototype experiments were conducted for three different pipeline wall thicknesses under three pressure resistant conditions. The experimental test results and simulation analysis results are shown in Fig 21, where the horizontal axis is the pipeline wall thickness, the vertical axis is the ultimate pressure, the blue bar block is the simulation analysis result, and the orange bar block is the experimental test result.

From the figure, it can be seen that with the increase of pipeline wall thickness, the pressure resistance capacity is enhanced. The simulation analysis results are close to the experimental test results, with the same trend of change and an error of less than 5%. Therefore, the simulation analysis method and results are verified to be reasonable and reliable, proving the correctness of the simulation analysis for pipe end strengthening.

## 6. Conclusion

This study addresses the weaknesses in the connection areas of long-distance pipelines, particularly focusing on the issue of weld strength at the pipe ends. It proposes a novel method for reinforcing the pipe ends using Arc Welding Additive Manufacturing technology. After analyzing the ellipticity of the pipe ends and leveraging AWAM to optimize geometry and mechanical properties, the strength and stiffness of the pipeline connection areas are significantly improved. The main findings of the research are as follows:

(1) Analysis of the Relationship Between Pipe End Ellipticity and Stress-Strain.Taking finite element simulation as a tool, the study systematically analyzes how pipe end ellipticity affects stress and strain. Variations in the ellipticity of the pipe end directly influence stress distribution and deformation characteristics at the pipeline connection. Pipe ends with larger ellipticity are more prone to stress concentration, increasing the risk of failure at these connection points.
(2) Optimization of Pipe End Additive Remanufacturing Process.An additive manufacturing technology simulation model was established for the pipe end remanufacturing process. By comparing the results of finite element analysis with analytical methods, the study demonstrates that additive manufacturing effectively corrects ellipticity errors, optimizes geometry, and enhances the strength and stiffness of the pipeline connection areas. The fillet radius selection is critical for controlling stress distribution; a large radius can lead to stress concentration, compromising the pipeline’s load-bearing capacity.
(3) Optimization Design and Finite Element Analysis Based on Ansys.Taking Ansys software as a tool, finite element analysis was conducted for the optimization of pipe end design, considering parameters such as thickening height, length, and fillet shape. A mathematical model was developed to determine the optimal design parameters, which include a thickening height of 10 mm, a thickening length of approximately 800 mm, and a fillet radius of 15 mm. These optimized design parameters significantly enhance the compressive and tensile strength of the pipeline connection areas, ultimately improving the overall reliability and safety of the pipeline.

Overall, the method proposed in this study offers a novel approach to optimizing pipeline connection areas by utilizing Arc Welding Additive Manufacturing technology for reinforcing pipe ends. This approach not only improves the geometry and corrects ellipticity errors, but also enhances the overall strength and stiffness of the pipeline, significantly contributing to the reliability and safety of the pipeline system. The pipeline material selected in this paper is common alloy steel, and future research can cover a variety of materials such as X80/X100 pipeline steel and stainless steel, and provide different reinforcement schemes for different material choices. Environmental experiments of pipelines were carried out to simulate alternating conditions of high and low temperatures or combined with partial corrosive media to evaluate the fatigue life of pipelines with different materials under different environments, thereby promoting the application of AWAM technology in pipeline repair and remanufacturing.

## Supporting information

S1 File Minimal data set. (PDF)
