# Supplementary material for: The research on enhancing the strength and durability of pipeline connection ports using arc welding additive manufacturing
Source: PLoS One. 2025 Jun 4;20(6):e0324598. doi: 10.1371/journal.pone.0324598 (PMC12136347; doi:10.1371/journal.pone.0324598)
Supplement: S1 File — (PDF) [file pone.0324598.s001.pdf]

Minimal data set

1. Pipeline geometric parameters

The geometric parameter data of the pipe model used in the simulation analysis example are as follows: the outer diameter of the pipe is 1219mm, the length is 3000mm, and the wall thickness is 20mm.

2. Simulation working condition parameters

The mesh partitioning method for the finite element simulation analysis selects multiple regions, and the mesh size is 15mm.

The selection of the internal pressure of the pipeline referred to the ASME B31.8 pipeline standard, and its data was 6.4Mpa.

The fixed support is selected at the end of the pipe on the unreinforced and thickened side.

3. Optimize the variation range of design parameters

The variation range of the thickness parameter for pipe end reinforcement and thickening is 10mm-15mm.

The variation range of the length parameter for pipe end reinforcement and thickening is 50mm-1200mm.

The variation range of the fillet radius parameter for pipe end reinforcement and thickening is 5mm-20mm.

4. Test results of pipeline pressure resistance verification

The data of the pipeline pressure resistance test and simulation are shown in Table 1.

Table 1 Comparison of Pipeline Pressure resistance Test and Simulation Results

Pipe wall thickness/mm	Experimental result/MPa	Simulation result/MPa
2.5	10.7	11.0
2.7	12.1	11.7
3.0	14.1	13.4
